# Unravelling the Mystery of Vestibular Paroxysmia: A Case Series

**DOI:** 10.7759/cureus.99269

**Published:** 2025-12-15

**Authors:** Venkatesh Manchikanti, Prachet Kulakarni, Manjiri Bapat, Bobby Jose

**Affiliations:** 1 Radiology, Medcare Hospital Sharjah, Sharjah, ARE; 2 Neurology, NMC Royal Hospital, Sharjah, ARE; 3 Radiology, NMC Royal Hospital, Sharjah, ARE; 4 Neurosurgery, Medcare Hospital Sharjah, Sharjah, ARE

**Keywords:** dizziness, tinnitus, vertigo, vestibular paroxysmia, vestibulocochlear nerve

## Abstract

Vestibular paroxysmia (VP) is a neurological disorder characterised by sudden-onset, recurrent, short-term episodes of vertigo and imbalance, often resulting from neurovascular conflict between a vascular loop and the vestibulocochlear nerve (VIII-cranial nerve). We present four cases of VP, highlighting the clinical presentation, diagnostic workup including magnetic resonance imaging (MRI), and medical management. Case 1: A 40-year-old female; MRI of the brain and internal auditory canal showed a loop of the anterior inferior cerebellar artery (AICA) coursing between the facial and vestibulocochlear nerves on the left, consistent with neurovascular conflict. Case 2: A 44-year-old male; MRI revealed a loop of the AICA indenting the left VII/VIII cranial-nerve complex at the entrance of the left internal auditory canal. Case 3: A 37-year-old female; MRI demonstrated a vascular loop at the right cerebellopontine angle, indicating neurovascular conflict. Case 4: A 39-year-old male; MRI showed a vascular loop in the left cerebellopontine angle causing modest compression of the left VII/VIII cranial-nerve complex along its posterosuperior aspect. These cases underscore the importance of high-resolution imaging in identifying neurovascular compression in VP, as well as early recognition and treatment with sodium-channel-blocking agents (carbamazepine, oxcarbazepine), which have shown efficacy in reducing attack frequency and intensity. Given the overlapping clinical features of VP with other episodic vestibular syndromes, awareness of characteristic imaging findings and therapeutic response is essential for accurate diagnosis and optimal management.

## Introduction

Vestibular paroxysmia (VP) is a rare disorder of the balance system characterised by recurrent episodes of vertigo. Its aetiology is attributed to vascular compression of the vestibulocochlear nerve. Among the various causes of dizziness in adults, benign paroxysmal positional vertigo (BPPV) is the most prevalent (17.1%), followed by psychogenic dizziness (15%), central vestibular syndromes (12.3%), and migraine-associated vertigo (11.4%). Less common aetiologies include vestibular neuritis (8.3%), bilateral vestibulopathy (Dandy syndrome) (7.1%), and VP (3.7%) [1,2].

VP represents a unique form of peripheral vertigo arising from a neurovascular conflict (NVC) at the root entry zone of the vestibulocochlear nerve [3]. The attacks are brief, typically lasting less than one minute, but may occur in clusters, sometimes exceeding 30 episodes per day [4]. The vertigo associated with VP is generally recurrent, paroxysmal, and of a spinning nature, with episodes lasting from seconds to several minutes.

In approximately 95% of cases, VP results from compression of the vestibulocochlear nerve (cranial nerve VIII) by the anterior inferior cerebellar artery (AICA), while the posterior inferior cerebellar artery (PICA) accounts for about 5% of cases [5,6]. Rarely, compression by the vertebral artery has also been reported [7].

Auditory symptoms often accompany VP, including tinnitus and, less frequently, sensorineural hearing loss. Visual manifestations, such as nystagmus, often provoked by hyperventilation, and oscillopsia, may also occur [8]. Brand et al. further identified a correlation between VP and acrophobia (fear of heights) in 56% of patients [9]. Comprehensive diagnostic criteria for VP were established in the 2016 consensus statement of the Bárány Society [10].

## Case presentation

Case 1

**Figure 1:**
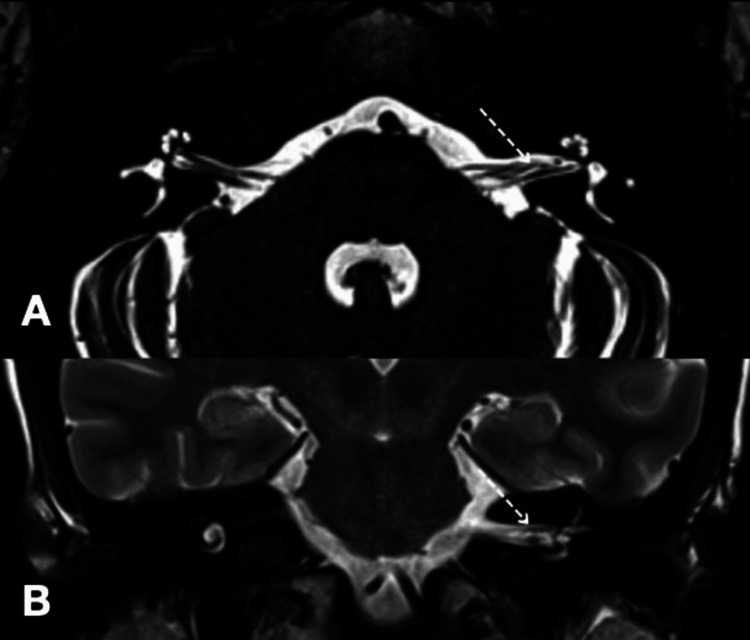
Magnetic resonance image analysis of Case 1 of constructive interference in steady state (CISS) sequence (A) and coronal T2 (B) showing a loop of the anterior inferior cerebellar artery (AICA) (dotted arrow) coursing between the facial nerve and vestibulocochlear nerve within the left internal auditory canal.

A 40-year-old woman presented with a two-month history of recurrent, severe spinning and non-spinning vertigo. The episodes were disabling, occurring multiple times a day, and led to a marked decline in her daily functioning and quality of life. They were occasionally accompanied by brief headaches, but she denied nausea, photophobia, visual disturbances, hearing loss, or tinnitus. Her hearing examination was normal. Her medical history included type 1 diabetes (on insulin since age 15), hypothyroidism, and dyslipidaemia. MRI of the brain and internal auditory canals excluded BPPV, vestibular neuritis, Menière’s disease, and vestibular migraine. High-resolution MRI demonstrated a loop of the AICA passing between the facial and vestibulocochlear nerves in the left internal auditory canal, consistent with an NVC. She was started on carbamazepine 400 mg/day, which was increased to 600 mg/day and eventually to 800 mg/day; following escalation, her condition improved and the frequency of attacks reduced significantly (Figure 1).

Case 2

**Figure 2:**
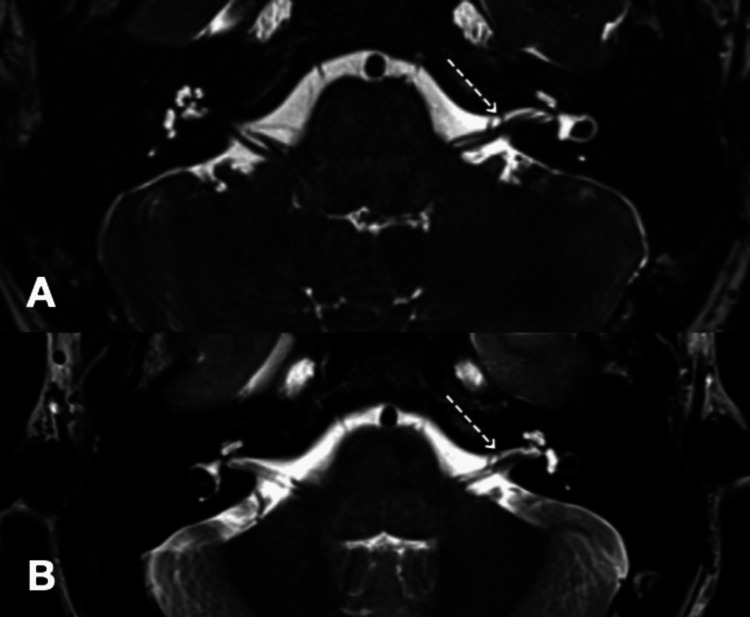
Magnetic resonance image analysis of Case 2 of constructive interference in steady state (CISS) sequence (A) and axial T2 (B) showing a vascular loop of the anterior inferior cerebellar artery (AICA) causing mild indentation over the left seventh and eighth cranial nerve complex (dotted arrow) at the entrance of the left internal auditory canal.

A 44-year-old man presented with a three-month history of recurrent, severe non-spinning vertigo, occurring many times daily and significantly impairing his daily functioning and quality of life. These brief episodes, often triggered by sudden changes in posture, mostly lasted under one minute, though a few persisted for several minutes. He reported no headache, hearing loss, or tinnitus, and both his vision and hearing were within normal limits. Neurological examination on one occasion revealed mild, unsteady gaze-evoked nystagmus. MRI of the brain and internal auditory canal revealed a loop of the AICA slightly indenting the left VII/VIII cranial-nerve complex at the entrance of the left internal auditory canal (Figure 2). The diagnosis of an NVC between the vestibulocochlear nerve and the AICA was considered, and a diagnosis of VP was made. He was initially treated with carbamazepine 600 mg daily, with symptomatic improvement; due to drowsiness, he was then switched to oxcarbazepine, after which his symptoms improved by more than 50%.

Case 3

**Figure 3:**
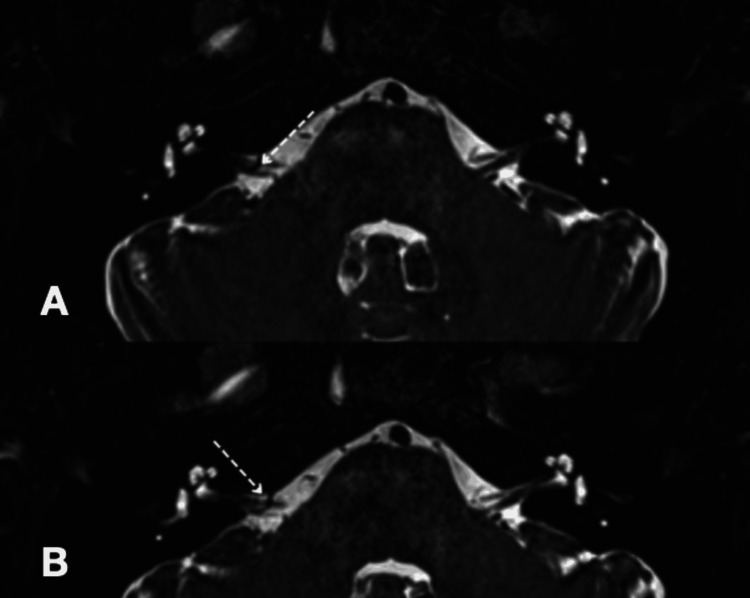
Magnetic resonance image analysis of Case 3 of constructive interference in steady state (CISS) sequence (A and B) showing a vascular loop at the right cerebellopontine angle causing mild compression and displacement of the seventh and eighth cranial nerve complex along the inferior aspect (dotted arrow).

A 37-year-old woman reported an eight-month history of brief episodes of giddiness, lasting from a few seconds up to one minute, often described as a sudden sensation of spinning or being pushed. These occurred in bursts of four to five episodes per day for several days, followed by periods with minimal or no attacks. Her hearing and vision were normal, and she did not experience tinnitus. She also reported occasional neck discomfort and generalised body aches, which were considered unrelated to her vestibular symptoms. MRI of the brain revealed a vascular loop at the right cerebellopontine angle, producing slight compression and displacement of the VII/VIII cranial-nerve complex at its inferior aspect (Figure 3), consistent with an NVC. She was treated with oxcarbazepine 600 mg/day and showed more than 50% improvement within 10 days of therapy.

Case 4

**Figure 4:**
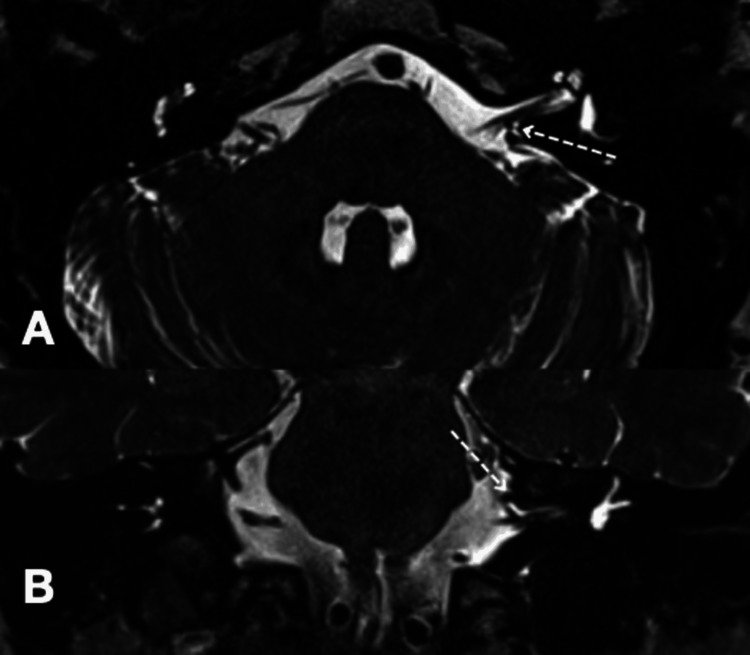
Magnetic resonance image analysis of Case 4 of constructive interference in steady state (CISS) sequence (axial A and coronal B) showing a vascular loop in the left cerebellopontine angle causing mild compression over the seventh and eighth cranial nerve complex along the posterosuperior aspect (dotted arrow).

A 39-year-old man reported several days of frequent episodes of giddiness, each lasting from a few seconds up to a minute, occurring multiple times per day. His symptoms were sometimes aggravated by changes in posture. MRI of the brain revealed a vascular loop at the left cerebellopontine angle, causing mild compression of the VII/VIII cranial-nerve complex along its posterosuperior aspect (Figure 4), suggesting an NVC. He was treated with oxcarbazepine 600 mg daily, resulting in significant improvement.

An informed consent form was signed by all the patients to publish this report.

## Discussion

The Bárány Society has proposed diagnostic criteria for VP. For definite VP, all of the following must be fulfilled: (a) at least 10 attacks of spinning or non-spinning vertigo; (b) each lasting less than one minute; (c) stereotyped phenomenology in that patient; (d) response to treatment with carbamazepine or oxcarbazepine; and (e) the clinical picture is not better explained by another diagnosis [5]. For probable VP, the criteria are slightly relaxed: at least five attacks, duration less than five minutes, spontaneous or head-movement provoked, stereotyped phenomenology, and not better accounted for by another diagnosis [11].

The pathophysiology of VP is thought to involve ephaptic (paroxysmal) inter-axonal transmission between adjacent, partly demyelinated axons of the vestibulocochlear (VIII) nerve. Because the root entry zone (proximal ~15 mm of the nerve) is still covered by central myelin (oligodendrocytes), it is particularly vulnerable to vascular compression [12]. The most frequent mechanism is focal irritation of the nerve by a pulsating blood vessel (commonly the AICA), leading to demyelination and generation of spontaneous discharges [13]. Compared to other cranial nerves, the VIII nerve has a relatively long central myelin portion, making it more susceptible to such NVC [5].

Differential diagnoses must include Menière’s disease, vestibular migraine, BPPV, superior semicircular canal dehiscence, panic attacks, and other episodic vestibular syndromes [12]. High-resolution MRI (e.g., constructive interference in steady state (CISS)/fast imaging employing steady-state acquisition (FIESTA) sequences) can help identify vascular loops in contact with the vestibulocochlear nerve, though the presence of a loop alone is not diagnostic because many healthy individuals also show such contact (some studies quote up to around 30-50 % of controls) [14].

Regarding treatment, a positive response to carbamazepine or oxcarbazepine lends strong support to the diagnosis. Medications, such as lacosamide, valproic acid, and phenytoin, have also been tried as alternatives. In one study of 29 patients, roughly 40 % achieved complete remission after stopping the anticonvulsant, and another 20% remained symptom-free for over one year [15]. For those who respond to medication but cannot tolerate it or where side-effects are problematic, microsurgical decompression (microvascular decompression) of the offending vessel-nerve conflict may be considered.

In our case series, all four patients fulfilled key aspects of the criteria: frequent brief vertigo attacks, stereotyped phenomenology, exclusion of other diagnoses, MRI evidence of a vascular loop in contact with the VII/VIII nerve complex, and positive response to carbamazepine/oxcarbazepine. This supports the diagnosis of definite VP. The imaging findings in our patients (vascular loops at the cerebellopontine angle or internal auditory canal involving the AICA) align with the literature showing frequent AICA involvement [16]. The therapeutic benefit observed in our patients underscores the importance of early identification and treatment to improve quality of life.

## Conclusions

Our cases highlight the importance of recognising this condition through careful clinical evaluation and correlating MRI findings with the patient’s response to carbamazepine or oxcarbazepine to establish an accurate diagnosis. Treatment with carbamazepine plays a central role in both confirming the diagnosis and effectively managing symptoms, significantly improving patients’ quality of life. Early diagnosis and appropriate therapy with carbamazepine or oxcarbazepine can substantially alleviate recurrent vertigo and imbalance, leading to marked improvements in overall health and daily functioning.
